# 1,25-Dihydroxyvitamin D Decreases Tertiary Butyl-Hydrogen Peroxide-Induced Oxidative Stress and Increases AMPK/SIRT1 Activation in C2C12 Muscle Cells

**DOI:** 10.3390/molecules24213903

**Published:** 2019-10-29

**Authors:** Eugene Chang

**Affiliations:** Department of Nutritional Science and Food Management, Ewha Womans University, Seoul 03760, Korea; eugenis77@hotmail.com; Tel.: +82-2-3277-4426

**Keywords:** adenosine monophosphate-activated protein kinase (AMPK), muscle, mitochondria, oxidative stress, sirtuin 1 (SIRT1), vitamin D

## Abstract

Enhanced oxidative stress has been associated with muscle mitochondrial changes and metabolic disorders. Thus, it might be a good strategy to decrease oxidative stress and improve mitochondrial changes in skeletal muscle. In the present study, we investigate the role of the most biologically active metabolite of vitamin D, 1,25-dihyroxyvitamin D (1,25(OH)2D) in oxidative stress and mitochondrial changes in tertiary butyl-hydrogen (tBHP)-treated C2C12 muscle cells. Differentiated C2C12 muscle cells were pretreated with tBHP, followed by 1,25(OH)2D for additional 24 h. An exogenous inducer of oxidative stress, tBHP significantly increased oxidative stress, lipid peroxidation, intracellular damage, and cell death which were reversed by 1,25(OH)2D in C2C12 myotubes. 1.25(OH)2D improves tBHP-induced mitochondrial morphological changes such as swelling, irregular cristae, and smaller size and number, as observed by transmission electron microscope. In addition, 1,25(OH)2D treatment increases mtDNA contents as well as gene expression involved in mitochondrial biogenesis such as PGC1α, NRF1, and Tfam. Significant increments in mRNA levels related to antioxidant enzymes such as Nrf2, HMOX1, and TXNRD1, myogenic differentiation markers including myoglobin, muscle creatine kinase (MCK), and MHCІ and ІІ, and vitamin D metabolism such as CYP24, CYP27, and vitamin D receptor (VDR) were found in 1,25(OH)2D-treated myotubes. Moreover, upon t-BHP-induced oxidative stress, significant incremental changes in nicotinamide adenine dinucleotide (NAD) levels, activities of AMP-activated protein kinase (AMPK)/sirtulin 1 (SIRT1), and SIRT1 expression were noted in 1,25(OH)2D-treated C2C12 muscle cells. Taken together, these results suggest the observed potent inhibitory effect of 1,25(OH)2D on muscle oxidative stress and mitochondrial dynamics might be at least involved in the activation of AMPK and SIRT1 activation in muscle cells.

## 1. Introduction

Oxidative stress—an imbalance between the production of reactive oxygen species (ROS) and antioxidant capacity—has been implicated in the pathophysiology of obesity and its-associated metabolic diseases including hypertension, hyperglycemia, and dyslipidemia [1,2]. Overproduction of ROS leads to irreversible modifications such as cellular injury and decreased antioxidant capacity in cellular components [3], which in turn contributes to mitochondrial dysfunction, characterized as diminished mitochondrial biogenesis, altered membrane potential, and decreased mitochondria number, and excessive oxidative production [4,5]. Reduced muscle mitochondrial content, mitochondrial function, and oxidative capacity has been reported in obesity and obesity-associated insulin-resistant and type 2 diabetic individuals [6,7]. Therefore, the decrease of ROS and the improvement of mitochondria biogenesis and function in skeletal muscle might be the potential targets for the prevention and/or treatment of metabolic complications.

An increasing body of evidence suggests vitamin D deficiency might be an indispensable factor for obesity and type 2 diabetes [8,9,10,11]. A close relationship between low vitamin D status and reduced muscle strength, mass, and function has been reported [12,13,14]. Several intervention studies demonstrate that vitamin D supplementation decreases the incidence of falls with enhanced muscle strength and muscular function [15,16,17]. In addition, vitamin D administration as 1,25-dihydroxyvitamin D (1,25(OH)2D) stimulates key pathways for muscle growth and differentiation by the direct regulation of nuclear vitamin D receptor (VDR) [18,19,20,21] and enhances mitochondrial function, dynamics, and enzyme function in human skeletal muscle cells [22]. In addition to VDR expression, 25-dihydroxyvitamin D3 24-hydroxylase (CYP24) levels involved in the degradation of 1,25-dihydroxyvitamin D (1,25(OH)2D), the most biologically active form of vitamin D and 25-hydroxyvitamin D3 1-alpha-hydroxylase (CYP27) expression related to the conversion from 25-hydroxyvitamin D to 1,25(OH)2D were found in C2C12 muscle cells [21]. In contrast to the favorable effects of vitamin D on muscle mass and function, vitamin D is associated with the increment of intramyocellular lipid contents and myocellular lipid partitioning, one of main contributor of ROS production [23,24,25]. Given close relationship between ROS and mitochondrial changes in muscle, molecular mechanisms by which vitamin D influences muscle mitochondrial biogenesis and function under oxidative stress need to be investigated.

Several lines of evidence demonstrates that critical energy sensors, adenosine monophosphate-activated protein kinase (AMPK) and sirtuin 1 (SIRT1) regulates the key transcripts such as peroxisome proliferative activated receptor gamma coactivator1α (PGC1α) and nuclear respiratory factor 1 (NRF1), which are associated with muscle mitochondrial biogenesis and function [26,27,28]. Apart from being energy regulators, both AMPK and SIRT1 are also implicated in redox sensing. In the regulation of cellular redox balance, SIRT1 acts as a potent intracellular inhibitor of oxidative stress by the deacetylation of transcription factors including nuclear factor erythroid 2-related factor (Nrf2) [29,30]. Upon enhanced stress levels, a critical antioxidant sensor, Nrf2 is dissociated from sequestration complex and translocated to the nucleus where a transcription factor, Nrf2 interacts with antioxidant-responsive element (ARE). It leads to the transcriptional activation of its target genes such as heme oxygenase-1 (HMOX1), thioredoxin reductase 1 (TXRND1), NAD(P)H-quinone oxidoreductase 1 (NQO1), superoxide dismutase, glutathione peroxidase, and glutathione S-transferase [31]. There is the crosstalk between Nrf2 and AMPK activation, which provides the protection against oxidative challenge [32,33]. Hence, the AMPK/SIRT1 signaling axis is a promising therapeutic target for oxidative stress and its-associated mitochondrial changes, which are sequentially implicated to metabolic complications. In the current study, the protective effect of vitamin D on aberrant mitochondrial changes in C2C12 muscle cells under oxidative stress induced by tertiary butyl-hydrogen peroxide (tBHP).

## 2. Results

### 2.1. Effect of 1,25(OH)2D on Cell Viability

**Figure 1:**
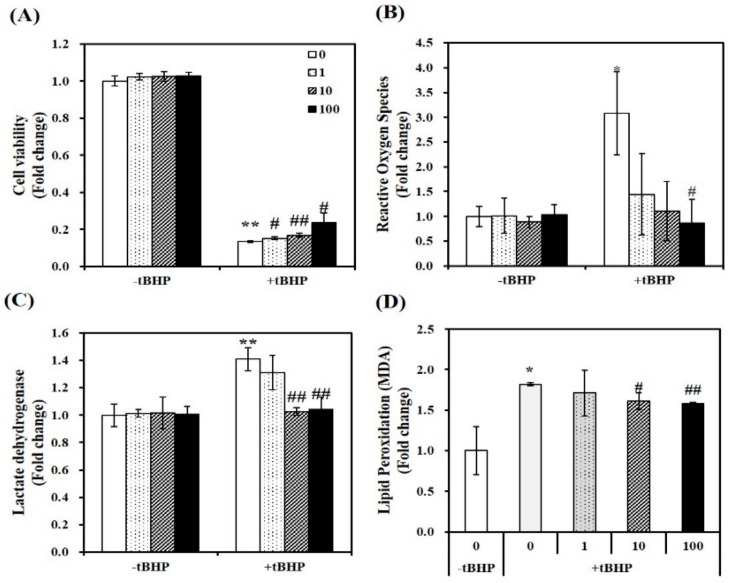
Effect of 1,25-dihydroxyvitamin D (1,25(OH)2D) on cell viability, oxidative stress, lipid peroxidation, and cellular damage in tertiary butyl-hydrogen peroxide-treated C2C12 muscle cells. On day 6 after differentiation induction, C2C12 muscle cells were incubated with tBHP (0.1 mM, 24 h) and treated with 1,25(OH)2D for additional 24 h in a dose-dependent manner (0, 1, 10, or 100 nM). The dissolved blue formazan (**A**), reactive oxygen species (ROS) (**B**), lactate dehydrogenase (LDH) levels (**C**)**,** and lipid peroxidation (MDA) (**D**) were measured and expressed as the fold change compared to vehicle control. Data are expressed as mean ± SEM. Experiments represent at least two or three independent experiments (*n* = 6–14 per group). * *p* < 0.05, ** *p* < 0.01 compared to vehicle control. ^#^ *p* < 0.05; ^##^ *p* < 0.01 compared to tBHP control.

In tBHP-treated C2C12 myotubes, the cytotoxicity of 1,25(OH)2D was analyzed by 3-(4,5-dimethylthiazol-2-yl)-2,5-diphenyltetra-zolium bromide (MTT) assay, which measures mitochondrial dehydrogenase activity, as described previously [33]. A concentration of tBHP at 100 μM induced an 83% reduction in cell viability (*p* < 0.01). In tBHP-incubated muscle cell, 1,25(OH)2D stimulated cell viability in a dose-dependent manner, started at a dose of 1 nM. At a dose of 100 nM 1,25(OH)2D, a 1.78-fold maximal increase was found in Figure 1A.

### 2.2. 1,25(OH)2D Reduces Oxidative Stress, Cellular Damage, and Lipid Peroxidation in tBHP-Incubated C2C12 Muscle Cells

To investigate the role of 1,25(OH)2D in tBHP-induced oxidative stress, intracellular ROS was determined with 2′,7′-dichlorofluorescin diacetate (DEFCA), as previously mentioned [34]. Treatment of tBHP showed a significant 3.08-fold increment in intracellular ROS, as compared to the control cells (*p* < 0.05). 1,25(OH)2D dose-dependently reduced ROS levels in tBHP-incubated myotubes, as shown in Figure 1B. Next, intracellular damage was measured as LDH contents. Incubation of tBHP induced a 1.23-fold increase in LDH contents, which was dose-dependently inhibited by 1,25(OH)2D, with a significant increment evident at 10 nM (*p* < 0.01) (Figure 1C). In addition, lipid peroxidation was analyzed by measuring malondialdehyde (MDA) levels, as an end product of lipid peroxidation. In C2C12 muscle cells, tBHP treatment induced a 1.82-fold increase of MDA contents, which was suppressed by 1,25(OH)2D in a dose-dependent manner (Figure 1D). These results illustrate that 1,25(OH)2D restores tBHP-increased ROS, lipid peroxidation, cell damage, and death.

### 2.3. Influence of 1,25(OH)2D on Mitochondrial Size and mtDNA Contents in C2C12 Mytotubes

**Figure 2:**
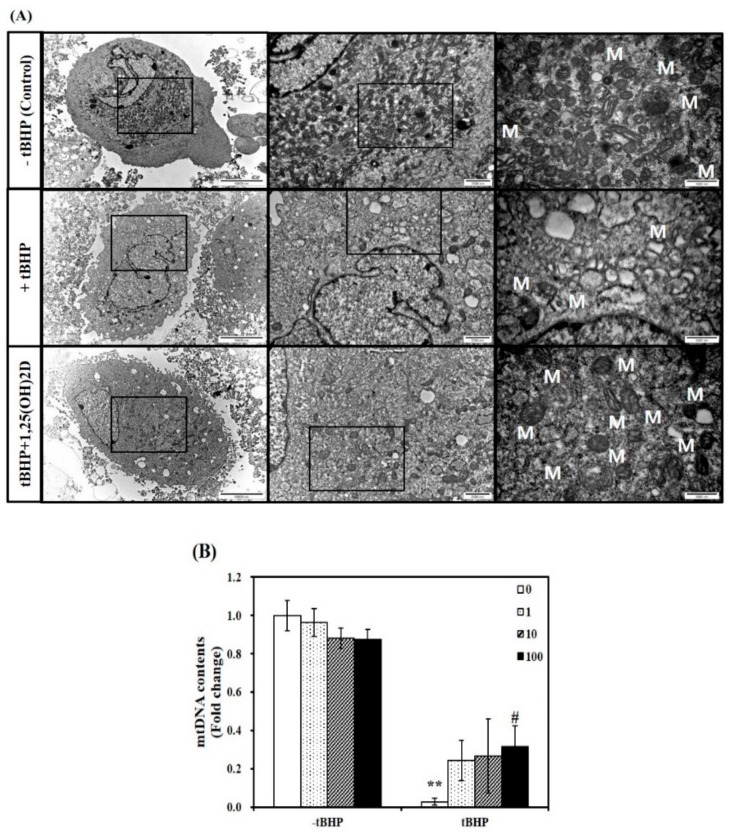
Influence of 1,25-dihydroxyvitamin D (1,25(OH)2D) on mitochondrial morphology and mitochondrial DNA (mtDNA) contents. Transmission electron microscopy (magnification of 7000, 20,000, and 50,000; scale bars = 10, 2, and 1 μm) (**A**). M demonstrates the position of mitochondria. The mtDNA contents were quantified by qRT-PCR (**B**). Results are expressed as mean ± SEM from two independent experiments (*n* = 6 per group). ** *p* < 0.01 compared to vehicle control. ^#^ *p* < 0.05 compared to tBHP control. Control (-tBHP), vehicle-treated C2C12 muscle cells; + tBHP, 0.1 mM tertiary butyl-hydrogen peroxide-treated myotubes; tBHP + 1,25(OH)2D, pretreatment with 0.1 mM tBHP following 1,25(OH)2D incubation (100 nM, 24 h).

Next, a TEM analysis was executed to investigate mitochondria morphological changes in C2C12 muscle cells. Compared to C2C12 control cells, tBHP-treated cells showed significant mitochondrial structural and morphological changes, as evidenced by irregular cristae and swollen mitochondria in accompanied with smaller size and number of muscle mitochondria. These unfavorable mitochondrial morphology changes were inhibited by 1,25(OH)2D treatment (Figure 2A). To determine whether mitochondria morphological change are related to mitochondrial biogenesis, mitochondrial DNA (mtDNA) contents were measured. The mtDNA levels in tBHP group were significantly lower than that in the control group (*p* < 0.01). In a dose-dependent manner, 1,25(OH)2D treatment increased the mtDNA contents in C2C12 muscle cells. A 11-fold maximal mtDNA level was found at a level of 100 nM 1,25(OH)2D, compared to tBHP (Figure 2B).

### 2.4. Gene Expression Involved in Mitochondrial Biogenesis, Antioxdiant Enzymes, Myogenesis, and Vitamin D Metabolism in C2C12 Muscle Cells

**Figure 3:**
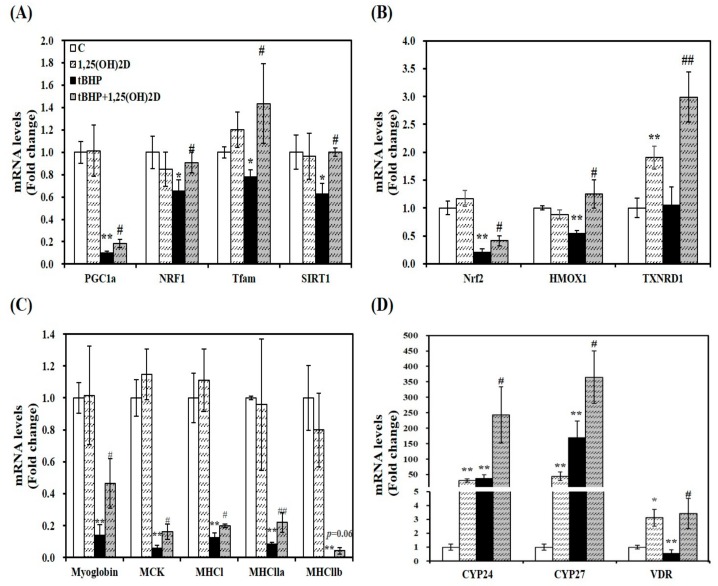
Effects of 1,25-dihydroxyvitamin D (1,25(OH)2D) on gene expression involved in mitochondrial biogenesis (**A**), antioxidant enzymes (**B**), myogenesis (**C**), and vitamin D metabolism (**D**). mRNA levels were determined by RT-PCR and normalized for all samples to β-actin. The value of each bar represents mean ± SEM. Experiments represent two independent experiments (*n* = 8 per group). * *p* < 0.05, ** *p* < 0.01 compared to vehicle control. ^#^ *p* < 0.05, ^##^ *p* < 0.01 compared to tBHP control.

As a previous study, mitochondrial oxygen consumption, volume, and dynamics are regulated by 1,25(OH)2D [22]. To investigate the effect of 1,25(OH)2D on mRNA levels related to mitochondrial biosynthesis, mRNA expression of PGC1α, NRF1, and mitochondrial transcription factor A (Tfam) was analyzed by quantitative real-time polymerase chain reaction (qRT-PCR). Treatment of tBHP significantly suppressed mRNA levels of PGC1α, NRF1, and Tfam, compared to C2C12 control cells. 1,25(OH)2D treatment (100 nM, 24 h) significantly upregulated PGC1α, NRF1, and Tfam mRNA expression by 1.8, 1.4, 1.8, and 1.6-fold, respectively in tBHP-incubated muscle cells, (Figure 3A). These results suggest that 1,25(OH)2D-induced transcriptional changes may underlie the 1,25(OH)2D-induced mitochondrial changes upon tBHP-induced oxidative stress in C2C12 muscle cells.

Next, a master regulator of antioxidant response, Nrf2 and its downstream gene expression was analyzed by qRT-PCR to delineate how 1,25(OH)2D treatment reversed tBHP-increased oxidative stress and lipid peroxidation. 1,25(OH)2D incubation significantly increased mRNA levels of Nrf2, HMOX1, and TXNRD1 by 2.1, 2.3, and 2.8-fold, respectively, compared to tBHP-treated C2C12 myotubes (Figure 3B).

A close association between myogenesis and ROS production led to measure mRNA expression of muscle differentiation markers. Compared to vehicle-treated control cells, tBHP significantly decreased mRNA levels of myoglobin, muscle creatine kinase (MCK), and muscle-specific markers such as myosin heavy chain I (MHCI), MHCIIa, and MHCIIb (*p* < 0.01), which were reversed by 1,25(OH)2D treatment (Figure 3C).

As shown in Figure 3D, 1,25(OH)2D treatment significantly increased CYP24 and CYP27 mRNA transcripts regardless of tBHP treatment. Compared to the vehicle control, VDR mRNA expression was significantly suppressed by tBHP treatment (*p* < 0.01). 1,25(OH)2D significantly upregulated VDR mRNA level by 6.1-fold in tBHP-treated C2C12 muscle cells (*p* < 0.05).

### 2.5. 1,25(OH)2D Stimulates SIRT1 and AMPK Activities in tBHP-Treated C2C12 Myotubes

**Figure 4:**
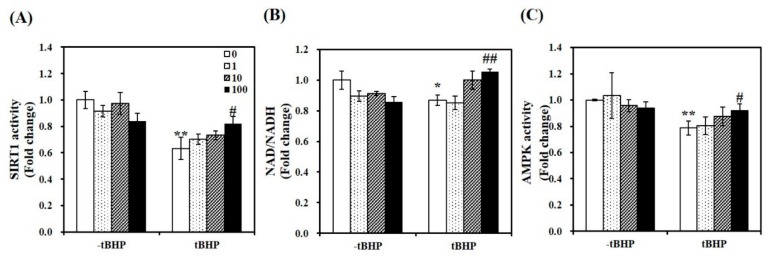
Effects of 1,25-dihydroxyvitamin D (1,25(OH)2D) on AMP-activated protein kinase (AMPK) /sirtulin 1 (SIRT1) activities and the ratio of nicotinamide adenine dinucleotide (NAD) to NADH. AMPK activity (**A**), SIRT1 activation (**B**), and NAD/NADH (**C**) were analyzed by commercial kits, normalized to their relative protein levels, and expressed as the fold change compared to vehicle control. The value of each bar represents mean ± SEM. Experiments represent at least two or three independent experiments (*n* = 8–10 per group). * *p* < 0.05, ** *p* < 0.01 compared to vehicle control. ^#^ *p* < 0.05, ^##^ *p* < 0.01 compared to tBHP control.

To demonstrate whether 1,25(OH)2D-induced mitochondrial biosynthesis is involved in SIRT1, we measured mRNA expression and activity of SIRT1. As shown in Figure 3A, 1,25(OH)2D-treated C2C12 showed a 1.59-fold increase in SIRT1 mRNA expression in tBHP-supplemented muscle cells (*p* < 0.05). Similar to SIRT1 mRNA levels, tBHP significantly suppressed SIRT1 activity by 37%, compared to the control cells (*p* < 0.01). 1,25(OH)2D incubation for 24 h significantly increased t-BHP-induced downregulation of SIRT1 activity in a dose-dependent manner. With statistical difference, 1,25(OH)2D (100 nM)-treated C2C12 muscle cell had a 1.29-fold increment of SIRT1 activity (Figure 4A). In regard to the results demonstrating 1,25(OH)2D-increased SIRT1 mRNA expression and activity, the ratio between nicotinamide adenine dinucleotide (NAD) and NADH was determined. In same line of the decrement in SIRT1 gene expression and activity, tBHP significantly inhibited the NAD to NADH ratio by 13%, when compared to the control cells (*p* < 0.05). A 1.21-fold increment in the ratio of NAD/NADH was measured in 1,25(OH)2D-treated C2C12 muscle cells at a dose of 100 nM (*p* < 0.05) (Figure 4B). Furthermore, we investigated whether 1,25(OH)2D influences AMPK activity in relation to enhanced SIRT1 activity and transcripts. As shown in Figure 4C, tBHP treatment significantly reduced AMPK activity by 21% in C2C12 cells, which were dose-dependently reversed by 1,25(OH)2D treatment.

## 3. Discussion

Mitochondria are involved in essential cellular functions such as energy metabolism, ATP generation, intracellular calcium regulation, ROS production, cellular survival and death. Mitochondrial dysfunction and impaired antioxidant capacity in skeletal muscle has been implicated as key mechanisms for the pathophysiology of obesity and obesity-associated health complications [1,2,4,5]. Therefore, it is critical to prevent oxidative stress and its-related mitochondrial dynamics for the prevention and/or treatment of obesity and its-related comorbidities. In the present study, we investigated the effect of 1,25(OH)2D, the most active metabolite of vitamin D on oxidative stress and mitochondrial changes in differentiated C2C12 muscle cells.

Several lines of evidence demonstrate that vitamin D supplementation alleviates local and systemic oxidative stress and inflammation in rodents and humans [35,36,37,38]. In addition, the negative association between vitamin D and the incidence of obesity and its-related type2 diabetes has been reported [8,10]. A role of vitamin D in muscle has been implicated in muscle strength and fall incidence [15,16,17]. In muscle cells, 1,25(OH)2D stimulates growth and differentiation and improves muscle mass and function [18,19,20], however, the effect of 1,25(OH)2D on muscle oxidative stress and mitochondrial changes has never been investigated. In the present study, tBHP having better stability was utilized as a model of exogenous oxidative stress [39]. Significant increments in MDA levels, a final product of lipid peroxidation, intracellular ROS, cellular injury, and cell death were observed by tBHP, which were reversed by 1,25(OH)2D in C2C12 myotubes. Consistent with previous studies showing the inverse association between vitamin D and oxidative stress [35,36], these results demonstrate the beneficial effects of vitamin D on oxidative stress in C2C12 muscle cells.

The process of muscle differentiation including cell cycle arrest, increased expression of myotube-specific genes and cell fusion is a highly organized and tightly regulated by muscle-specific transcription factors [40]. In matured fiber-type muscle, several muscle-specific genes including MyHC and the major structural protein in myotubes such as myoglobin, and MCK are predominantly expressed [41]. The formation of multinucleated myotubes has been associated with mitochondrial proliferation, a decrease in ROS production, and an enhanced dependence on oxidative phosphorylation in a positive manner of PGC transcription via AMPK activation [42,43]. When vitamin D was administered in a form of 1,25(OH)2D during the process of C2C12 muscle differentiation, inconsistent results have been reported [18,44,45]. In differentiated C2C12 muscle cells, no further effect of 1,25(OH)2D on differentiation was not noted [44]. In relation to vitamin D and muscle differentiation, VDR deletion inhibits myogenic differentiation of C2C12 cell line [46]. In addition, vitamin response genes such as CYP24 and CYP27 were expressed in muscle cells [21]. In the current study, mRNA levels related to myogenesis and vitamin D metabolism were measured. 1,25(OH)2D significantly upregulated gene expression involved in muscle differentiation and mature muscle type such as myoglobin, MCK, MHCI, and MHCIIa and mRNA levels related to vitamin D metabolism including CYP24, CYP27, and VDR in C2C12 myotubes. Given a close association between myogenesis and ROS production, 1,25(OH)2D-decreased ROS might be associated with enhanced gene expression involved in muscle differentiation and vitamin D metabolism. Still, further investigation is needed to measure 1,25(OH)2D-induced changes in ROS levels and muscle differentiation in siVDR-transfected C2C12 muscle cells by using precise methods such as an observation of mytobutes appearance using photomicrograph, fluorescence-activated cell sorting (FACS), and fluorescence microscopy.

There is a reciprocal link between mitochondrial morphology and ROS generation. Defects in mitochondria such as impaired oxidative capacity and antioxidant defense result from overproduction of oxidative stress and ROS-mediated lipid peroxidation, which sequentially inhibits membrane fluidity and mitochondrial mass and function [3]. Indeed, chronical ROS augmentation decreases mitochondrial contents in skeletal muscle [47]. Decreased mitochondrial contents and function has been reported in skeletal muscle tissues from obese humans [6]. Thus, mitochondrial dynamics might be used as a potential therapeutic target for treatment. There are several transcription factors involved in mitochondrial biogenesis and function. Increased PGC1α expression is involved in the synthesis of nuclear respiratory factors (NRFs), all of which upregulates Tfam, a major transcription factor for mtDNA transcription [48,49]. The relationship between increased Tfam transcription and enhanced replication and transcription of mtDNA contents demonstrates that mtDNA seems to be linked to mitochondrial mass [50]. A significant increase of mRNA levels involved in mitochondrial biogenesis such as PGC1α, NRF1, and Tfam was found by 1,25(OH)2D in tBHP-treated C2C12 muscle cells. In our *in vitro* cell culture system, an exogenous source of ROS, t-BHP induced profound mitochondrial morphological changes such as swelling and disarrayed cristae with decreased size and number of mitochondria, observed by TEM. In addition, mtDNA contents was significantly reduced by tBHP incubation. In 1,25(OH)2D-treated C2C12 muscle cells, larger size and increased number of mitochondria was observed by TEM, in accompanied with increased transcript of mtDNA, an indicator of mitochondrial mass. These data suggest that 1,25(OH)2D-decreased lipid peroxidation, cellular injury, and cell death might be associated with reduced ROS production and mitochondrial biogenesis.

In response to oxidative stress, Nrf2, a transcription factor scavenges ROS and confers cellular protection by upregulating antioxidant enzymes such as HMOX1, TXNRD1, NQO1, and, superoxide dismutase [31]. Disruption of Nrf2 enhances mitochondrial oxidative stress, lipid peroxidation, and apoptosis in skeletal muscle [51,52]. One of key transcripts for mitochondrial biogenesis, NRF1 promoters contains multiple AREs, which is interacted with translocated Nrf2 upon ROS induction [53]. Indeed, decrements in mitochondrial biogenesis and function are found in Nrf2 KO muscle [54]. It suggests a role of Nrf2 in muscle mitochondrial dynamics as a cellular defense mechanism against stress condition. In addition, AMPK signaling protects ROS by regulating Nrf2 translocation to ARE promoter via SIRT1-mediated deacetylation [29,32,33]. Therefore, AMPK and SIRT1 play pivotal role in redox sensor. AMPK activation is involved in muscle mitochondrial biogenesis and function by regulating key transcription factors such as PGC1α and NRF [26]. The involvement of AMPK in PGC1α-mediated mitochondrial biogenesis might be the key for protecting mitochondrial dysfunction and ROS production [55]. An AMPK activator, resveratrol protects oxidative stress-induced heart muscle damage, which is abolished by AMPK inhibition using dominant negative AMPK constructs [56]. Reciprocally, AMPK stimulates SIRT1 activation by modulating intracellular NAD levels [57]. In addition, SIRT1 controls post-translational modification of PGC1α by deacetylation and modulates PGC1α activation, which inhibits transcription factors involved in the relation of cellular redox balance [28,29,30]. In the present study, AMPK activation and Nrf2 and its downstream gene levels such as HMOX1 and TXNRD1 were significantly increased by 1,25(OH)2D in tBHP-treated C2C12 myotubes. tBHP-induced reduction in mRNA expression and activation of SIRT1, a potent intracellular inhibitor of oxidative stress and inflammatory response was reversed in 1,25(OH)2D-treated C2C12 muscle cells. Moreover, the ratio of NAD to NADH was increased by 1,25(OH)2D in tBHP-treated mytobutes. Thus, the redox balance and mitochondrial biosynthesis by 1,25(OH)2D might be associated with the activities of AMPK and SIRT1, in relation to NAD/NADH ratio. Based on two reports illustrating VDR-dependent changes in human muscle function and enzymes [22] and the involvement of SIRT1/AMPK cascade in adipocyte oxidative stress and antioxidant defense mechanism [58], there is a possibility that the favorable effects of 1,25(OH)2D on tBHP-induced oxidative stress in C2C12 myotubes might be a VDR and/or SIRT1 dependent. Therefore, a following study using muscle-specific targeted VDR and SIRT1 modification might be warranted.

To the best of our knowledge, the present study, for the first time, demonstrates that 1,25(OH)2D ameliorates tBHP-induced oxidative stress, lipid peroxidation, cell damage, and death, as well as mitochondria appearance and mtDNA levels in C2C12 myotubes. In addition, 1,25(OH)2D treatment significantly increases mRNA levels involved in mitochondrial biogenesis, antioxidant enzymes, and muscle differentiation and NAD-associated AMPK/SIRT1 activities in tBHP-treated C2C12 muscle cells. These results suggest that maintaining or improving vitamin D status might be good strategy to prevent muscle oxidative stress and its-relevant health diseases.

## 4. Materials and Methods

### 4.1. Cell Culture and Treatment

Mouse C2C12 myoblast cell line was obtained from ATCC (American Type Culture Collection; Manassas, VA, USA) and maintained in Dulbecco’s Modified Eagle Medium (DMEM; Gibco, Grand Island, NY, USA) supplemented with 10% fetal bovine serum (FBS; Gibco), 100 U/mL of penicillin, and 100 mg/mL of streptomycin (Gibco) in a humidified atmosphere of 95% air and 5% CO_2_ at 37 °C. At 90% confluence, myoblasts were induced to differentiate in DMEM with 2% horse serum (Gibco), 100 U/mL of penicillin, and 100 mg/mL of streptomycin (Gibco). On day 6 after differentiation, muscle cells were pretreated with tBHP (0.1 mM; Sigma-Aldrich, St. Louis, MO, United States) for 24 h, then with 1,25(OH)2D (Sigma-Aldrich) for an additional 24 h. 1,25(OH)2D was dissolved in absolute ethanol and treated at the given concentrations as indicated.

### 4.2. Cell Viability Assay

Cell viability was determined by MTT colorimetric assay to estimate mitochondrial function as described previously [33]. At the end of treatment, 5 mg/mL MTT (Sigma) dissolved in DMEM was added to fresh medium at a final concentration and incubated for 1 h at 37 °C. After the solution was removed, the formed blue formazan was dissolved in DMSO and the absorbance was measured at 570 nm by a Varioskan plate reader (Thermo Scientific, Waltham, MA, USA). Cell viability was calculated as the fold change with respect to the vehicle control without tBHP or 1,25(OH)2D treatment.

### 4.3. Measurement of Intracellular Reactive Oxygen Species (ROS)

The measurement of intracellular ROS content was performed by using 2′,7′-dichlorofluorescin diacetate (DCFDA), as described before [34]. C2C12 myotubes were pretreated with tBHP, followed by 1,25(OH)2D in a dose-dependent manner. After washing cells with PBS (pH 7.4), differentiated C2C12 cells were incubated with 20 μM DCFH-DA in serum-free DMEM, an oxidation-sensitive dye for 45 min at 37 °C. The amount of intracellular ROS formation was determined by a fluorometric measurement at 485 nm/535 nm (excitation/emission).

### 4.4. Determination of Lactate Dehydrogenase (LDH) Levels

An indicator of cell damage, the concentrations of lactate dehydrogenase (LDH) were analyzed using a commercially available kit (Abcam, Cambridge, MA, USA). C2C12 myotubes were prepared in a cold LDH assay buffer and centrifuged for 15 min at 4 °C at 10,000× *g* to remove any insoluble materials. In an assay, LDH reduces NAD to NADH, which reacts with the specific probe to generate an intense color product at 450 nm. Protein was determined using a bicinchoninic acid (BCA) protein assay kit (Thermo Scientific). LDH contents were normalized by their respective protein levels and expressed as the fold change compared to C2C12 control cells.

### 4.5. Lipid Peroxidation Assay

Malondialdehyde (MDA) contents, an end product of lipid peroxidation, were quantified using a colorimetric assay kit (Abcam). In this lipid peroxidation assay protocol, the MDA in the sample reacts with thiobarbituric acid (TBA) to produce a MDA-TBA adduct. This adduct can easily be quantified colorimetrically at 532nm. Briefly, C2C12 myotubes were prepared in a lysis buffer containing butylated hydroxytoluene, having antioxidant properties to stop further sample peroxidation. Insoluble materials were removed after centrifugation at 13,000 × *g* for 10 min at 4 °C. Levels of lipid peroxidation were normalized to their respective protein concentrations, as determined by a BCA protein assay kit (Thermo Scientific).

### 4.6. RNA Isolation, Reverse Transcription and Quantitative Real-Time Polymerase Chain Reaction (qRT-PCR)

Total RNA was isolated using a RNeasy Mini Kit (Qiagen, Valencia, CA, USA) following the manufacturer’s instructions. Isolated RNA was subsequently reverse-transcribed to cDNA using a MMLV Reverse Transcriptase Kit (Bioneer, Daejeon, Korea) and incubation at 37 °C for 60 min followed by incubation at 95 °C for 5 min using GeneAMP^®^ PCR system 2700 (Applied Biosystems, Foster City, CA, USA). mRNA expression was determined by qRT-PCR as described previously [38]. Primer sequences used are provided in Table S1. mRNA level of each target gene was normalized to the housekeeping gene, β-actin and expressed as the fold change relative to C2C12 control cells.

### 4.7. Transmission Electron Microscopy (TEM)

Differentiated C2C12 myoblasts were washed with PBS and then collected for centrifugation at 1000× *g* for 3 min at 4 °C. The supernatant was discarded, and cells were fixed with 2.5% glutaraldehyde plus paraformaldehyde in a 0.1 M phosphate buffer (pH = 7.4) for 2 h at room temperature and TEM assay was performed following a previous study [59].

### 4.8. Determination of Mitochondrial DNA (mtDNA) Levels

DNA was extracted using a genomic DNA extraction kit (Bioneer) and mtDNA levels were calculated using real-time qPCR by measuring the mitochondrial gene (COX1, subunit 1 of cytochrome oxidase) versus the nuclear gene (GAPDH, glyceraldehyde 3-phosphate dehydrogenase), as described previously [60].

### 4.9. SIRT1 Activity Assay

A SIRT1 activity assay kit (Abcam) were used for the measurement of SIRT1 activity. According to the manufacturer’s protocol, extracted nuclear fractions were executed to measure SIRT1 activity in the presence of NAD and a production of fluoro-substrate peptides was detected using a fluorometric microplate reader at 340 nm/460 nm (excitation/emission). Results was normalized to their respective protein concentrations and expressed as the fold change compared to control cells.

### 4.10. Measurement of AMPK Activity

AMPK activity was measured using an AMPK kinase assay kit (MBL International Co., Woburn, MA, USA). C2C12 mytobutes were incubated with anti-phospho-mouse IRS-1 S789 monoclonal antibody, which can be phosphorylated by AMPK and horseradish peroxidase (HRP)-conjugated anti-mouse IgG. AMPK activity was detected at a wavelength of 450 nm, normalized to their respective protein concentrations, and expressed as the fold change compared to control cells.

### 4.11. Determination of NAD to NADH Ratio

A NAD/NADH assay kit was utilized for the measurement of a ratio of NAD to NADH (Abcam), according to the manufacturer’s instructions. C2C12 muscle cells were snapped and frozen in liquid nitrogen and extracted at 4 °C with extraction buffer provided with the NAD/NADH assay kit. The ratio between NAD and NADH were normalized by their respective protein concentrations

### 4.12. Statistical Analysis

Data was analyzed by one-tailed Student’s t-test using SPSS Statistics 20 (SPSS, Inc., Chicago, IL, USA). Statistical significance was defined as *p* < 0.05. Data are expressed as mean ± standard error of the mean (SEM).

## 5. Conclusions

In conclusion, the present study demonstrates that 1,25(OH)2D significantly prevented tBHP-induced ROS, lipid peroxidation, cellular damage, and cell death in C2C12 muscle cells. A TEM observation illustrates that tBHP inhibited size and number of mitochondria with irregular cristae and swelling and decreased mtDNA levels, which was reversed by 1,25(OH)2D in myotubes. In tBHP-treated muscle cells, mRNA expression involved in mitochondrial biogenesis such PGC1α, NRF1, and Tfam, antioxidant defense such as Nrf2, HMOX1, and TXNRD1, myogenic differentiation including myoglobin, MCK, and MHCI and II, and vitamin D metabolism such as CYP24, CYP27, and VDR, were upregulated by 1,25(OH)2D. Moreover, 1,25(OH)2D significantly stimulated tBHP-decreased activities of AMPK and SIRT1 with a NAD/NADH ratio and SIRT1 expression. Hence, these results demonstrate that 1,25(OH)2D might have beneficial effects on ROS-induced muscle mitochondrial changes and might be applicable as the therapeutic potential for the prevention and/or treatment its-associated health complications

## Supplementary Materials

The following are available online at https://www.mdpi.com/1420-3049/24/21/3903/s1, Table S1: Primers used for quantitative real-time polymerase chain reaction (qRT-PCR).
