# Supplementary material for: 1,25-Dihydroxyvitamin D Decreases Tertiary Butyl-Hydrogen Peroxide-Induced Oxidative Stress and Increases AMPK/SIRT1 Activation in C2C12 Muscle Cells
Source: Molecules. 2019 Oct 29;24(21):3903. doi: 10.3390/molecules24213903 (PMC6864759; doi:10.3390/molecules24213903)
Supplement: Supplementary file 1 [file molecules-24-03903-s001.docx]

**Table S1.** Primers used for quantitative real-time polymerase chain reaction (qRT-PCR)

| **Gene** | **GeneBank no.** | **Forward sequence (5’-3’)** | **Reverse sequence (5’-3’)** | **Product size (bp)** |
| --- | --- | --- | --- | --- |
| β-actin | NM_007393.5 | GGACCTGACAGACTACCTCA | GTTGCCAATAGTGATGACCT | 208 |
| CYP24 | NM_009996.4 | AGT GAA CCT GTG GAG ATG CTG | AGT TGT CCC ATG CTC TGG TC | 201 |
| CYP27 | NM_010009.2 | GTG GGC TCA GTG TTT GTG TC | CAT ATC CTCCTC AGG CTT TC | 189 |
| HMOX1 | NM_010442.2 | CACGCATATACCCGCTACCT | CCAGAGTGTTCATTCGAGCA | 175 |
| MCK | NM_007710.2 | CAT GGA GAA GGG AGG CAA TA | GAC GAA GGC GAG TGA GAA TC | 233 |
| MHCI | NM_080728.3 | TCG AGG CTT CTG GAA GTT GT | GGC ATT GAG TGG ACC TTC AT | 189 |
| MHCIIa | NM_001039545.2 | ATA CCA GCG CTT CCT TCT CA | GTC TAA GGC CAA GGG AAA CC | 182 |
| MHCIIb | NM_010855.3 | GGA AAA CTC GCC TGA CTC TG | GGA GAA GAG CGA GCT GAA GA | 207 |
| Myoglobin | NM_013593.2 | AAGGGCTCAGAGGACCTGAA | TGTGATTGGGCTAGAGGCTG | 119 |
| NRF1 | NM_001164226.1 | AAGTATTCCACAGGTCGGGG | TGGTGGCCTGAGTTTGTGTT | 238 |
| Nrf2 | NM_010902.4 | CTCGCTGGAAAAAGAAGTGG | CCGTCCAGGAGTTCAGAGAG | 240 |
| PGC1α | NM_008904.2 | GGGCCAAACAGAGAGAGAGG | GTT TCG TCC GAC CTG CGT AA | 250 |
| SIRT1 | NM_019812.3 | TACCCCATGAAGTGCCTCAA | AACCAATTCCTTTTGTGGGC | 203 |
| Tfam | NM_009360.4 | GAGGCCAGTGTGAACCAGTG | GCTCTGAAGCACATGGTCAA | 158 |
| TXNRD1 | NM_015762.2 | CAGCGAGGAGACCATAGAGG | GCACATTGGTCTGCTCTTCA | 155 |
| VDR | NM_009504.4 | CTA GAGGTG TTC GGC AAT GA | AGG AGA AAG GAT GAG CCA GA | 187 |

CYP24, 1,25-dihydroxyvitamin D(3) 24-hydroxylase; CYP27, 25-Hydroxyvitamin D3 1-alpha-hydroxylase; HMOX1, heme oxygenase 1; MCK, muscle creatine kinase; MHC, myosin heavy chain; NRF1, nuclear respiratory factor 1; Nrf2, nuclear factor erythroid 2-related factor 2; PGC1α, peroxisome proliferative activated receptor gamma coactivator 1α; SIRT1, sirtuin 1; Tfam, mitochondrial transcription factor A; TXNRD1, thioredoxin reductase 1; VDR, vitamin D receptor

| 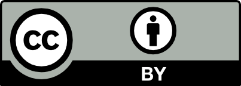 | © 2019 by the authors. Submitted for possible open access publication under the terms and conditions of the Creative Commons Attribution (CC BY) license (http://creativecommons.org/licenses/by/4.0/). |
| --- | --- |
